# Carbohydrate-Mimetic Peptides for Pan Anti-Tumor Responses

**DOI:** 10.3389/fimmu.2014.00308

**Published:** 2014-06-30

**Authors:** Thomas Kieber-Emmons, Somdutta Saha, Anastas Pashov, Behjatolah Monzavi-Karbassi, Ramachandran Murali

**Affiliations:** ^1^Department of Pathology and Winthrop P. Rockefeller Cancer Institute, University of Arkansas for Medical Sciences, Little Rock, AR, USA; ^2^Stephan Angelov Institute of Microbiology, Bulgarian Academy of Sciences, Sofia, Bulgaria; ^3^Research Division of Immunology, Department of Biomedical Sciences, Cedars-Sinai Medical Center, Los Angeles, CA, USA

**Keywords:** glycans, carbohydrate-mimetic peptide, mimotope, vaccines, structural design, cancer

## Abstract

Molecular mimicry is fundamental to biology and transcends to many disciplines ranging from immune pathology to drug design. Structural characterization of molecular partners has provided insight into the origins and relative importance of complementarity in mimicry. Chemical complementarity is easy to understand; amino acid sequence similarity between peptides, for example, can lead to cross-reactivity triggering similar reactivity from their cognate receptors. However, conformational complementarity is difficult to decipher. Molecular mimicry of carbohydrates by peptides is often considered one of those. Extensive studies of innate and adaptive immune responses suggests the existence of carbohydrate mimicry, but the structural basis for this mimicry yields confounding details; peptides mimicking carbohydrates in some cases fail to exhibit both chemical and conformational mimicry. Deconvolution of these two types of complementarity in mimicry and its relationship to biological function can nevertheless lead to new therapeutics. Here, we discuss our experience examining the immunological aspects and implications of carbohydrate–peptide mimicry. Emphasis is placed on the rationale, the lessons learned from the methodologies to identify mimics, a perspective on the limitations of structural analysis, the biological consequences of mimicking tumor-associated carbohydrate antigens, and the notion of reverse engineering to develop carbohydrate-mimetic peptides in vaccine design strategies to induce responses to glycan antigens expressed on cancer cells.

## Introduction

Among the most challenging of antigen targets for vaccine design are glycans (1). They are ubiquitous in nature and can be considered as one of the unique antigens expressed across pathogens and cancer cells. Glycans are fundamental to the biological functions of cell–cell communication, cell proliferation, and differentiation, and they mediate cell attachment, as well as mediating pathogen attachment and infection. Cancer cells, in particular, are noted for their aberrant glycosylation profiles that affect the metastatic process. Consequently, certain carbohydrate forms profoundly affect both the pathophysiology of infection and neoplasia (Table 1). A unique advantage in targeting tumor-associated carbohydrate antigens (TACAs) is that multiple proteins and lipids on cancer cells can be modified with the same carbohydrate structure which might be shared with bacterial antigens (2). Thus, targeting TACAs has the potential to broaden the spectrum of antigens recognized by the immune response, thereby lowering the risk of developing resistant tumors due to the loss of a given protein antigen.

**Table 1:** **Glycosphingolipid Constituents Shared Among Bacteria and Tumor cells**.

GSL series type	Structure	Bacterial species
Lacto	Galβ1 → 4GlcNAcβ1 → 3Galβ1 → 4Glcβ1 → Cer	*N. gonorrhoeae*
		*N. meningitidis*
		*Moraxella catarrhalis*
		Helicobacter pylori
		*H. influenzae*
		Campylobacter jejuni
		*H. ducreyi*
Globo	Galα1 → 4Galβ1 → 4Glcβ1 → Cer	*N. gonorrhoeae*
		*N. meningitidis*
		*H. influenzae* type b
		*H. influenzae* NT
		*B. catarrhalis*
Ganglio	GalNAcβ1 → 4Galβ1 → 4Glcβ1 → Cer	*N. gonorrhoeae*
	Galβ1 → 3GalNAcβ1 → 4Galβ1 → 4Glcβ1	
	GalNAcβ1 → 3Galβ1 → 4GlcNAcβ1 → 3Galβ1 → 4Glcβ1 → Cer	

*Lacto series, neolactoseries, Globosides, and Ganglioside antigens are found on tumor cells (3, 4) and on LOS of multiple bacteria (5–7)*.

We have come to learn that the manner a TACA is expressed will dictate how an immune effector mechanism will be invoked (8). Antibodies against glycolipids and globular glycoproteins are found to mediate complement-dependent cytotoxicity (CDC) because they extend less than 100 angstroms from the cell membrane while antibodies to mucins that extend up to 5000 angstroms from the cell surface do not (8). But TACAs are also associated with cell signaling activities whereby anti-TACA antibodies are capable of direct induction of cell death of number of tumor cell lines, but this activity has not been investigated in great detail (9, 10). In this context, TACAs are pan-targets on tumor cells because they are collectively and intimately involved in cell-death signaling pathways. Strategies that target TACAs have, therefore, potential clinical benefit as cell-death therapies. Anti-TACA antibodies can mediate significant reprograming of signaling events, with profound anti-tumor activities. The ability to induce antibodies reactive with multiple TACAs is relevant as heterogeneity of antigen expression in different cancers of the same type, as well as different cells of the same cancer, and heterogeneity of immune response in different patients make it likely that maximal anticancer effect may not result from immunization against a single antigen.

The success of carbohydrate-based vaccines against pathogens has led to technological advances in vaccine design, but they have typically been developed as mono or singular vaccine types requiring a polyvalent formulation to induce responses across carbohydrate types (11). While glycans are diverse in expression patterns and in their composition, the structural commonalities among glycans provide a template to target, at least some of them collectively, by directing the immune response toward these commonalities. Therefore, it is logical to target glycans in vaccine design, which can lead to the interruption of disease processes (11).

Among potential technological strategies is using carbohydrate-mimetic peptides (CMP) to induce responses to glycans on pathogens and cancer cells (12). Peptides can substitute as immunogens to target pathways involving protein–carbohydrate interactions and in carbohydrate-specific immunological reactions. However, there is a noted distinction between the ideas of antigenic mimicry versus the ability of a mimic to induce a response cross-reactive with a carbohydrate/glycan moiety.

Antigenic mimicry, in simple terms, is when one ligand competes with another for antibody binding. The origin of cross-reactivity involves thermodynamic and structural interpretations (13–15). The notion of immunological mimicry is less precise. Does it mean that the mimic generates the same antibody subset as the nominal antigen or just that it induces a response that cross-reacts with the nominal antigen?

Early on CMPs were shown to function as antigenic mimics (16, 17) but more importantly they were shown to induce serum antibodies in a variety of systems, having utility in directing responses to cancer cells and against pathogens (18–26). Most of all, unlike carbohydrate antigens, CMPs can prime for memory responses to TACAs (27) suggesting that the CMPs facilitate cognate interactions between B cells and T cells, which is something that carbohydrates/polysaccharides do not facilitate, but surrogate antigens of carbohydrates such as anti-idiotypic antibodies and CMPs should and can do. CMPs are not only a functional strategy to induce carbohydrate-reactive responses, but also they can function as probes to understand the structural basis for the dual recognition properties of antibodies, lectins, and T cells (12, 14, 15, 28, 29). Understanding the structural requirements for antibody and T-cell recognition provides a basis for identifying potentially new sets of immunogens that may have both fundamental immunological and clinical value. However, it has been argued that translation of such information into viable vaccines is still a long way off (30–32). Here, we briefly discuss the various perspectives and elements of CMPs useful to translate them into the clinic in tumor vaccine design applications to target glycans.

## Molecular Mimicry at a Glance

Molecular mimicry is now firmly considered as the basis of many autoimmune disorders, proposed as a pathogenic mechanism for autoimmune disease, as well as a probe useful in uncovering its etiologic agents (33). On the other hand, self-limiting autoimmunity may underlie some of the pathogenic mechanisms in infectious disease. This hypothesis is based in part on observed cross-reactivity of immune reagents with host “self” antigens and microbial determinants (33). Molecular mimicry is also suggested as a means to regulate immune homeostasis and to elicit responses against target antigens as evidenced by studies on anti-idiotypic antibodies (34). This model suggests that conventional T-cell/B-cell collaboration can explain communication between complementary idiotype [Id(+)] and anti-Id antibody at the cellular level that integrates present and previous data on B-cell regulation. Furthermore, this model provides a tool to probe carbohydrate immunology paradigms because the synergistic interaction of effector T and B cells require common recognition of identical tumor-associated antigen(s) (35). Anti-idiotypic antibodies have been proposed to mimic carbohydrate antigens and have been tested in the clinic (36–40).

**Figure 1:**
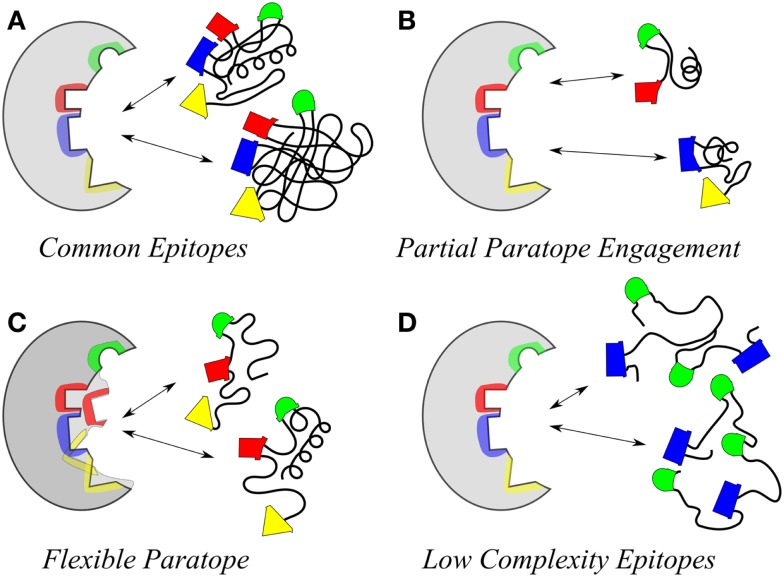
**Illustrative models highlighting the polyspecificity or cross reactivity of antigens for an antibody**. **(A)** Two different molecules may carry the same structure. **(B)** The same paratope may accommodate multiple smaller epitopes in different parts. **(C)** The flexibility of the paratope may allow for interaction with different epitopes. **(D)** Different flexible molecules with repetitive low complexity structure containing common groups (e.g., sugars) have a high probability of fitting in the same paratope. These are aspects of polyspecific binding, which are partially related (like **A** and **D**) and sometimes may occur in combination (**C** and any one of the rest).

At one level, an explanation for molecular mimicry is when a foreign antigen shares sequence or structural similarities with self-antigens. But on another level what defines the recognition and interaction basis for antigenic mimicry that ties to a functional immune response? Molecular mimicry in the context of antibody–antigen recognition is interpreted at several levels (Figure 1). The work by Hoffmuler et al. (41) suggests that a common epitope can be preserved among an ensemble of peptide variants. They demonstrated that the binding modes of intermediate conformation of selected peptides were characterized using complete sets of substitution analogs, revealing that a number of sequential substitutions accumulated without changing the pattern of key interacting residues. At a distinct step, however, one single amino acid exchange induces a change in the binding mode, indicating a flip in specificity and conformation (41). Regions of proteins with biased amino acid composition [so-called Low-Complexity Regions (LCRs)] are abundant in the protein universe (42). LCR-containing proteins tend to have more binding partners across different networks than proteins that have no LCRs. LCRs may be involved in flexible binding associated with specific functions, but also that their positions within a sequence may be important in determining both their binding properties and their biological roles (42).

Intrinsically disordered regions of proteins have also been associated with molecular mimicry (43), indicating the potential of highly flexible peptides as mimics. Such peptides may be attractive to induce pan anti-tumor responses, due to their potential ability to mimic multiple TACAs *in situ*. However, the structural diversity inherent to such peptides makes defining the precise nature of their mimicry of any or multiple TACAs even more challenging. Geometrical shape complementarity, the “lock and key” hypothesis, between antigen–antibody interaction, has long dominated immunological thinking. However, studies demonstrating the existence of a large number of monoclonal antibodies that can bind to a variety of totally unrelated self and foreign antigens (i.e., polyreactive antibodies) have modified this view. Consequently the lock and key model has been supplemented with an explanation focusing on the flexibility of antibody binding sites that can change conformation to accommodate different antigens (44).

**Figure 2:**
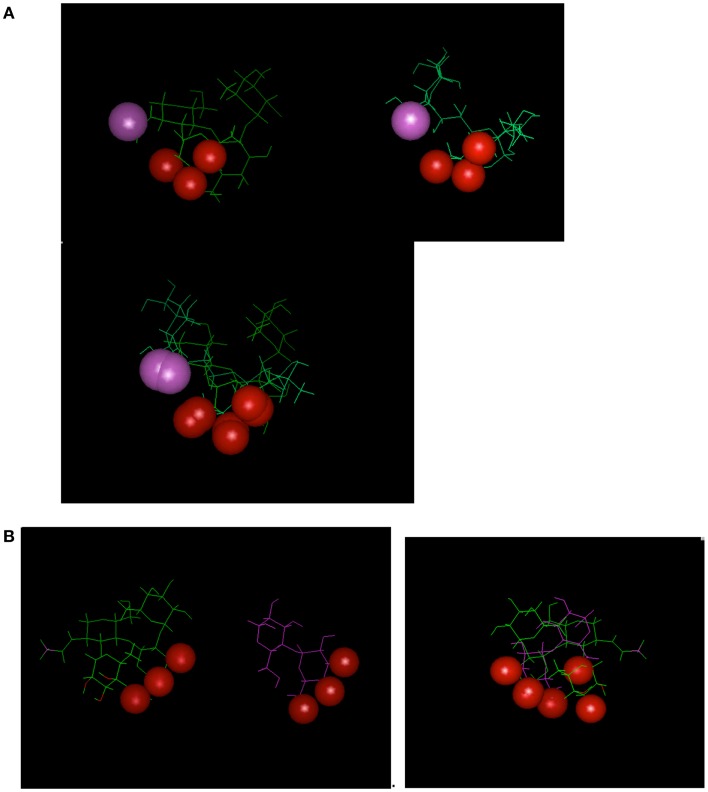
**Examples depicting similarity of epitopes in dissimilar carbohydrate antigens**. Epitopes (hydroxyls) are represented by red-spheres. **(A)** Relationship between Lewis Y antigen on left side of panel with MCP on right side. **(B)** Relationship between Lewis Y antigen on left side of panel with α1–4 Glucose on right side of panel. Interestingly the epitope defined on the glucose moiety defines a three-dimensional epitope on the Lewis Y antigen.

Antibodies induced by a CMP to the meningococcal group C capsular polysaccharide (18) were shown to be reactive with the Lewis Y antigen (20). Carbohydrate-reactive antibodies show the potential cross-reactivity for dissimilar carbohydrate forms that highlight the common epitope basis for cross-reactivity (Figure 2); Figure 2A shows that a common epitope is formed between α2–8 sialic acid and the neolactoseries antigen Lewis Y (18, 20). The potential of antibodies recognizing three hydroxyl groups might be cross-reactive with three hydroxyl groups displayed on two glycosyl groups (Figure 2B). This level of recognition leads to the idea that antibodies can recognize carbohydrate in the context of pan-recognition. The cases discussed above relate as much to the common epitope mechanism as to the low-complexity epitopes, which seems to be often the case in carbohydrate recognition.

What is discussed here, strictly speaking, is molecular interaction at the atomic level, while recognition is rather the system level processing of information relevant to immune function, i.e., self/non-self distinction and identification of previously met danger (32, 45). Specificity of interaction serves these purposes only in some aspects while others favor polyspecific binding. For T-cell receptors (TCR) antigen specificity is an emerging property of the system rather than a characteristic of the individual receptor (46). On a molecular level, TCRs are a rather promiscuous binder. Furthermore, in terms of pre-immune antibodies, polyspecificity has also the role of ensuring a complete repertoire. It funnels antigen/pre-immune antibody interactions into the somatic hypermutation process of refining specificity.

An interesting twist to this topic is the emerging notion of reverting specific antibodies to polyspecific binding or induced polyspecificity as a physiological mechanism operating for instance at the sites of inflammation (47–49). Yet, perhaps to most, typical polyspecific immune binding makes use of pattern recognition to generalize a danger context (50, 51). Functionally, the boundary between pattern recognition receptors and natural antibodies is fuzzy (52–54). Intrinsically, prone to polyspecificity by several mechanisms, antibody recognition of carbohydrates conceptually merges antigen and pattern recognition. In this regard, carbohydrate mimotopes (e.g., CMP) instead of mimicking one particular structure by another come about rather as mimics of patterns, not unlike synthetic TLR agonists. But carbohydrate mimotopes are not exclusively artificial. CMPs from natural proteins are known for some time (55, 56). Peptides from Mucin 1 cell surface receptor (MUC1) are the most interesting because they are considered mimics of the Gal-epitope (56). Natural peptides can adopt structures similar to carbohydrate antigens (21) and can exhibit binding kinetics similar to the nominal antigens that they mimic (21, 28).

Often times CMPs share no obvious consensus sequence but their amino acid sequences often contain aromatic and hydrophobic residues but also amino acids having cyclic side chains, including proline and glycine that affects the conformational properties of the mimic (13, 57). The predominance of aromatic residues in CMPs invokes interaction scenarios that include stacking and hydrophobic interactions. A basis for this is the notion that carbohydrate recognition by antibodies use hydrophobic faces on carbohydrate antigens (58). It is important to note that cohesive solvent–solvent interactions are the major driving force behind apolar association in solution (59). Consequently, interaction models that implicate important roles for dispersion forces in molecular recognition events should be interpreted with caution in solvent-accessible systems (59). In addition, other antibody recognition systems also suggest that dual antigen recognition could involve divergent antibody conformations of nearly equivalent energetic states (60). Therefore, developing high-affinity-binders might make use of antibody structural plasticity to mediate the recognition step without increasing the entropic cost (60).

### Humoral responses to CMPs

While a variety of CMPs have been developed with the ability to induce immune responses of desired specificities and functionality (61) they are perhaps most appealing as a probe to understand the immunological response to carbohydrate antigens. An important feature of CMPs is in their ability to mediate contact-dependent T-cell help as an obligatory role in humoral immune responses to T-cell dependent antigens. Cognate B-cell/T-cell interactions during the immune response to protein antigens depend on T-cell co-stimulation. Details of how such interactions govern immune responses to carbohydrate-conjugate vaccines are few. We have shown that immunization with CMPs activate peptide-specific T helper type 1 (Th1) and type 2 (Th2) responses (62, 63). However, while behaving like a Th1 antigen (63), multivalent peptide mimetics still could induce a high carbohydrate-reactive IgM/IgG ratio with an endpoint titer of 1:2,000 (20). These results suggest that the multiple antigen peptide form might function like a Th2 independent immunogen in BALB/c mice. Furthermore, we observed that CMPs mediate cognate B and T-cell interactions as CMPs can induce antibodies in a host with deficiency in IgM production that typically do not respond to carbohydrate antigens (62). In these studies apparently the B cells functioned as antigen-presenting cells. In addition, these studies suggest that B-cell subsets influence the interactions. More importantly, the type of TACA mimicked by the CMPs is expressed in mice (29, 64). Consequently, these studies are obtained in a toleragenic mouse model, further suggesting that tolerance is broken upon CMP immunization.

A characteristic of an effective mimotope based vaccine would be to prime for secondary responses upon boosting or challenge with native antigen (18, 27, 65–67). Peptide-mimotope anamnestic responses have been noted for mimotope-conjugates (65, 66). The identification of peptide mimetics relies upon the idea that antibody fine specificity epitope mapping patterns of carbohydrates and peptide mimetics might be used as a proxy for individual B-cell receptor specificity activated during a secondary antibody response. However, the idea of functional mimicry would suggest that immunization with a carbohydrate-mimic peptide might also induce a specific subset or restricted anti-carbohydrate response. Our studies indicate that since peptide-conjugates elicit immune responses in xid mice (62), it is likely that antibodies to peptide and carbohydrate immunogens might be structurally unique and derived from different antibody subsets.

### Potential for cellular immunity targeting carbohydrate antigens

Up until a few years ago, carbohydrate determinants were traditionally not considered as targets for Cytotoxic T-Lymphocytes (CTL) despite a variety of immunogenicity and specificity studies for the glycan moiety of synthetic *O*-glycosylated MHC-binding peptides suggest otherwise (68–70). GD2 was also implicated as a target upon CTL activation early on (71). Crystal structure analyses indeed show that T cells can recognize glycopeptides bound by MHC molecules on the surface of antigen-presenting cells (72, 73). T cells, therefore, have the potential to react with the carbohydrate moiety of neoglycopeptide antigens, suggesting that T cells can target carbohydrate antigens expressed on tumor cells. However, it is also possible to generate carbohydrate-specific unrestricted CTL responses with MHC class-I-binding carrier peptides (74) that might explain the GD2 response (71). Nonetheless, how such T-cell responses are generated is presently unclear. From a vaccine perspective, the construction of glycopeptide/protein immunogens is problematic.

Rather than simple molecular mimicry, unpredictable arrays of common and differential contacts on class-I complexes can be used for their recognition by the same TCR. For example, bacterial polysaccharides with a distinct charge-motif can be emulated by peptides that can activate T cells (75). Lysine–aspartic acid (KD) peptides with repeating units are able to stimulate CD4^+^ T cells *in vitro* and confer protection against abscesses induced by bacteria such as *Bacteroides fragilis* and *Staphylococcus aureus* (75). CMPs can induce a Th1 response in mice using a DNA platform (76). We have observed an augmented induction of CTL activity against Meth A tumor cells upon peptide-mimotope immunization (63, 77). The induction of carbohydrate-reactive T-lymphocytes with peptide mimics is based upon a functional definition of T-cell mimotopes. One possible explanation is that the peptide-mimotope activates CTLs, which bind to *O*-linked GlcNAc or GalNac glycopeptides associated with MHC Class-I. Based upon crystal structure analysis of MHC complexes with glycopeptides, it appears that the central region of the putative T-cell-receptor-binding site is dominated by the extensive exposure of the tethered carbohydrate (72, 73). Our modeling of CMPs in the MHC Class-I groove suggests that amino acids and glycans attached to a glycopeptides overlap in 3D space, providing an array of contacts for TCR recognition (12).

### Fidelity in mimicry

The ability to augment or enhance TACA-reactive antibodies using CMPs would be noteworthy. Much like anti-idiotypes, CMPs may elicit anti-saccharide responses, but fail to elicit the idiotypes and isotypes observed in the protective response to the microbial antigen (78). Functional antibodies depend not only on the host’s ability to mount an immune response, but also on its ability to mount the appropriate immune response. Whether an antibody response is protective or not may depend on both the fine antigenic specificity that may be associated with particular idiotypes and epitope binding characteristics, and the isotype, determining antibody effector function. Often times studies of peptide mimics selected by lectins or antibodies and then analyzed by structural approaches come to the conclusion that mimicry at structural level is minimal at best (13–15). The same conclusions are drawn in considering anti-idiotypic antibodies (79). Rather, mimics as peptides or anti-idiotypes serve as imprints of the structural characteristics of the nominal carbohydrate antigen and, consequently, give rise to antibodies with carbohydrate-like properties upon immunization. The question remains how to enhance the ability of TACA-mimetic peptides to induce TACA-reactive antibodies with higher titers and association constants. Herein lies the problem with mimics; the immune response is only assayed after a choice is made as to which mimic is to be followed. So what lessons can be learned about choosing the “true” mimic?

#### From lectins to vaccines

**Figure 3:**
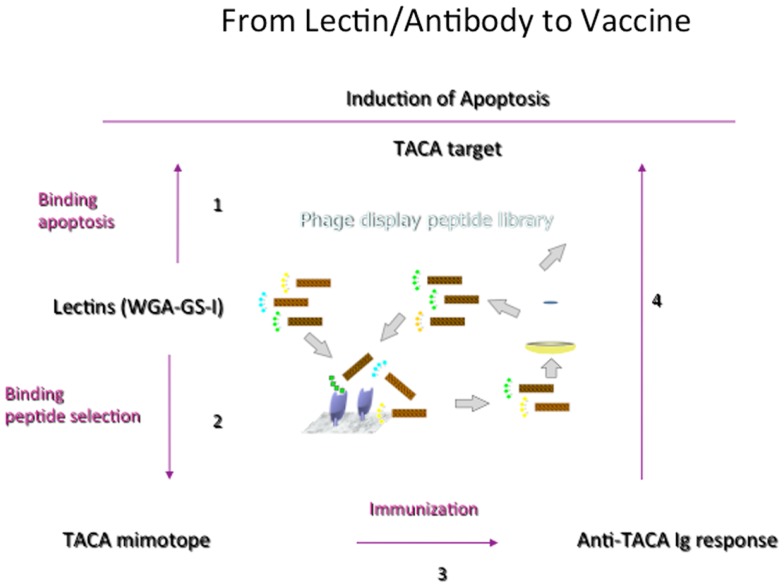
**General scheme of translating process of random phage library screening to functional vaccine**. Important to start with lectin or antibody with functionality but not all CMPs selected will induce the desired response. CMPs can be defined in a four-step process. (1) Lectins that trigger apoptosis of tumor cells are defined. (2) Biopanning against a random peptide display library identifies potential CMPs, which are confirmed by carbohydrate-peptide inhibition assays. (3) The potential of the CMPs to induce TACA-reactive antibodies is evaluated, as is (4) the ability of CMP-induced antibodies to mediate apoptosis of tumor cells.

While lectins have been generally used to identify CMPs and to understand the general features of recognition phenomena, Figure 3 outlines the general development of CMPs in vaccine design using lectins as a template to induce antibodies that would emulate the actions of lectins. We have shown that this concept can be brought into practice (80). Plant lectins like *Griffonia simplicifolia* lectin I (GS-1) and wheat germ agglutinin (WGA) mediate the apoptosis of tumor cells. We have investigated the possibility of using these lectins as templates to select peptide-mimotopes of TACAs as immunogens to generate cross-reactive antibodies capable of mediating apoptosis of tumor cells (80). Vaccine-induced anti-carbohydrate antibodies to both 106 and 107 (Table 2) reduced the outgrowth of micrometastases in the 4T1 spontaneous tumor model, significantly increasing survival time of tumor-bearing animals. This finding parallels suggestions that carbohydrate-reactive IgM with cytotoxic activity may have merit in the adjuvant setting if the right carbohydrate-associated targets are identified (81, 82). Interestingly, while both CMPs 106 and 107 are reactive with lectins only 107 induced responses that were directly cytotoxic to tumor cells. Both CMPS induced antibodies that mediated CDC, however, only CMP 107 induced serum IgM antibodies in mice that mediated the apoptosis of murine 4T1 and human MCF7 cell lines *in vitro*, paralleling the apoptotic activity of the lectins (80). This finding again highlights that selection of CMPs based upon antigenic mimicry does not automatically translate into inducing antibodies with a desired functionality.

**Table 2:** **Selected CMPs that we have studied**.

Peptide	Sequence	Lectin	Functionality
911	YRYRYGRYRSGSYRYRYGRYRSGS	Con A	Neutralizes HIV Lab isolates
912	RYRYGRYRSGS	Con A	
106	GGIYWRYDIYWRYDIYWRYD	GS-1, WGA	Mediates CDC
107	GGIYYRYDIYYRYDIYYRYD	GS-1, WGA	CDC, Apoptosis
P10	GVVWRYTAPVHLGDG	GS-1, WGA	Tumor growth inhibition
P10s	WRYTAPVHLGDG	GS-1, WGA	Tumor growth inhibition in mice, apoptosis in humans

Fundamental feature of these CMPs was their hydrophobic nature being built on motifs containing aromatic residues. Early on, peptides that mimic carbohydrate antigens were identified by analysis of reactivity of random peptide libraries on phage with the lectin Concanavalin A (ConA) (16, 17). These early peptides contain aromatic side chains, representing a generalized Trp/Tyr/X/Tyr (were X is a number of different residues) motif. Subsequent to these seminal studies other aromatic peptides displaying similarities to ConA-reactive ones were described (18, 83). Aromatic residues, hydrophobic, and hydrogen bonding amino acids seem favored but with the possibility that the W/YXY motif functionally mimic elements of Core 1 and 2 structures shared among otherwise dissimilar carbohydrate structures (Figure 2). Consequently, this motif type has been observed in peptides isolated by a number of anti-carbohydrate antibodies and lectins and might represent low-complexity surfaces (Figure 1) perhaps because of the bias in the amino acid composition of the mimetics. Such biased sequences do not necessarily converge on a canonical set of patterns although some motifs stand out. It is important to note how those peptides reactive with ConA were identified based upon a conception of antigen mimicry, as the work of Westerink et al. (18) were based upon immunological studies starting with an anti-Id that displayed immunological functionality.

#### Structure-based reverse engineering to discover peptide mimics

The caveats associated with screening libraries with either lectins or with antibodies often lead to identifying mimics that fail to mimic critical contacts that the carbohydrate makes with the protein, and there is a possibility that such peptides may bind to alternative sites on protein to the carbohydrate-binding site, making optimization of a true structural mimic from such a peptide impossible (84). The structural approaches to define the basis of mimicry have been previously discussed (13, 14). As mentioned above high-affinity peptides *per se* may not necessarily mimic critical contacts required for the function. In addition, the judicious choice of peptides for testing antibody responses against should be based on the peptide interaction with both the heavy and light chain in order to induce antibodies with similar antigen specific properties (28); as the combination of heavy and light chains will influence specificity. Thus, both the variable and the constant region of the antibodies induced by a peptide mimic or mimotope must be considered when assessing the success of any immunization.

To overcome the limitation of high-affinity peptides’ lack of immunological mimicry, we adopted a “reverse engineering approach” sometime ago, which places emphasis on the maintaining critical contacts between carbohydrates and its protein partner (28, 29). This method is similar to fragment-based drug discovery (28). We have previously reviewed the structural concepts and approaches used in vaccine design applications that illustrate the value and limitations of using chemical (peptide libraries which are mimics of a ligand) and immunological information to define novel peptide immunogens that function as mimotopes to generate immune responses targeting TACA (85) and glycans on the human immunodeficiency virus (86). In this context, we showed that concepts associated with pharmacophore design (now considered reverse engineering) could be used to define CMPs applied to vaccine design (21, 28). We demonstrated a structure-assisted vaccine design approach, whereby small molecules, defined in crystallographic databases, could be used in principle to define peptide mimetics emulating the three-dimensional interaction scheme of a native carbohydrate antigen (21, 28). More importantly, it was shown that virtual screening led to motifs being observed experimentally and that they could display binding energetics similar to the nominal carbohydrate antigen (28).

We have also shown that by using this approach, an immunogenic peptide (911 Table 2) can be designed *de novo* using ConA as a template inducing antibodies with the same functionality as ConA in neutralizing HIV isolates (21). In addition, we showed that peptides could adopt structures that are similar to carbohydrate conformations that include extended beta strand type and helical structures (21). Using reactivity patterns of glycan binding to ConA coupled with structural design concepts we identified a peptide (referred to as 911) (Table 2) that when rendered as a multiple antigenic peptide (MAP) was reactive with ConA at lower concentrations than those required for reaction of some native oligosaccharide ligands of ConA (21). The 911-MAP displayed competitive inhibition with carbohydrate ligands of ConA, indicating that it binds at an overlapping carbohydrate-binding site on ConA. Isothermal Calorimetric analyses and immunoprecipitation experiments suggest that a shorter monovalent putative peptide 912 (Table 2) exhibited a weak affinity comparable to that of MeαMan (21). The 911-MAP exhibited a higher association constant and free energy of association with ConA compared with that found upon binding of the putative 912 peptide and the Ka and ΔG values of 911-MAP are comparable to those of ConA-reactive trimannoside and pentasaccharide (21). Most importantly, the 911-MAP induce antibodies in mice that are capable of neutralizing HIV-1 III-B as assessed by p24 ELISA (21). This is work perhaps for the first time demonstrated that design-principles associated with CMPs could be useful to induce functional antibodies. Similar approaches have since been applied to investigate peptide recognition by anti-alpha-Gal antibodies (87) and in developing CMPs of gangliosides (88). As in our studies, it was found that peptides could interact with the same residues as those involved in carbohydrate recognition. In this context, CMPs are envisioned to be further enhanced as either inhibitors much like that in mainstream pharmacophore development or as in our case to develop vaccines targeting glycans.

To further emphasize the design principles to enhance the fidelity of mimicry, we tested the hypothesis that improving the hydrogen bond pattern through amino acid substitutions in a CMP, to be coincident with that for the carbohydrate ligand, will enhance the ability of CMPs to elicit anti-TACA antibodies with high titers and association constants (29). Based on anti-Id/Id crystal structures, highly directional bonds represent an important set of interactions to establish a basis for mimicry because they mainly confer the specificity in binding of the peptide and the carbohydrate antigen. In this exercise, we developed the CMP P10s (Table 2) (29). This CMP was identified from a random peptide library screen using the anti-GD2/GD3 antibody ME36.1 (89). P10 was shown to generate immune responses in mice that inhibited tumor growth *in vivo* (90).

In the development of P10s, we made use of the crystal structure of the anti-ganglioside antibody ME36.1 (29). Briefly, the crystal structure of ME36.1 was analyzed in the context of comparing GD2 binding and CMP binding using a molecular docking approach (29). Based on the hydrogen bonds interaction between GD2 and CDRs of ME36.1, P10s was designed. Conformational and docking calculations suggested that P10s would form an increased number of hydrogen bonds with ME36.1 that are in common with the GD2 hydrogen bond interaction pattern with ME36.1 [see Table 1 in Ref. (29)]. This increased level of mimicry would suggest that the immune response to GD2 upon immunization with P10s would be better. We observed that P10s did indeed induce higher titer antibodies to the target antigen and antigen expressing tumor cells than the parent CMP, P10. These studies suggest that for carbohydrate mimics, pharmacophore based design is superior over the conformational approach undertaken for other peptide mimics.

### Preclinical assessments of CMPs

Tumor-associated carbohydrate antigen are rather varied in their expression profiles on tumor cells and on normal tissue. TACAs are upregulated in many types of tumors, and therefore represent a potential vaccination target with widespread application. Cancer vaccines functionally resemble the process of autoimmune-mediated tissue damage (91).

Since tissue rejection is the goal of cancer immunotherapies, broad-spectrum, pan-antigens like TACA are plausible effective targets once the problem of their low immunogenicity is solved. This is the hope of CMP and anti-idiotypic vaccine research.

The basis of TACA mediate tumor rejection is akin to the observation that anti-Gal IgM and IgG mediate rejection of xenograft expressing α-gal glycoconjugates with terminal Galalpha1-3Galbeta1-4GlcNAc sequences (alpha-galactosyl epitopes, natural xenoreactive antigens) that are present on various tissues in pigs and are recognized by human anti-alpha-galactosyl (alpha-Gal) antibodies (92). The tissue-rejection mediated by α-Gal-reactive antibodies demonstrates the feasibility of targeting TACAs for tumor therapy because tumor-induced antibody responses resemble autoimmune responses (93).

The generation of tissue-rejection represents an important conceptual approach to cancer immunotherapy. Alpha-galactosylated xenoantigens (Galalpha1-3Galbeta1-4GlcNAcbeta1 and Galalpha1-3Galbeta1-4GlcNAcbeta1-3Galbeta1-4Glc) are often detected with the alpha-Gal-reactive lectin GS-1. However, this lectin exhibits a broad and variable specificity for carbohydrates terminating in alpha-Gal (94). The blood group reactive lectin GS-I, which recognizes alpha-galactosyl moieties is recognized as a surrogate marker to identify tumor expressed antigens reactive with anti-Gal antibodies and GS-I is of utility to interrogate terminal α-GalNAc/Gal expression on human tissues (95). Some of these antigens are also expressed on normal cells at low levels, potentially creating a state of immune tolerance.

We have previously demonstrated that vaccination with the CMPs 106 and 107 (Table 2) can induce antibody responses leading to cell-mediated cytotoxicity and apoptosis, respectively, in murine models of cancer (80). In preclinical studies, we observe that immunization in mice with these CMPs do not induce significant immunopathology, organs including liver, kidney, heart, lungs, intestines, stomach, lymph nodes, spleen, brain, spinal cord, and eyes were examined in H&E stained sections. These organs are reactive with GS-1 and the CMPs induce antibodies reactive with GS-1 antigens (29, 64). No significant cellular infiltrates were identified in any organ, including brain and spinal cord, from any animal, and there was no evidence of necrosis or extensive apoptosis in these sections (29, 64). It is likely that the level or pattern of expression of these molecules on the surface of tumor cells differs significantly from that on normal cells mediated by antibody avidity and the clustering of glycan epitopes (96). This difference in expression may account for the relative specificity of immunologic injury for tumor cells over normal cells.

Antibodies induced by CMPs are thought to have low affinities for TACA that might compensate for the low-affinity of the carbohydrate cross-reactive antibodies, minimizing the destruction of normal tissue. Such results demonstrate that repeated injections of CMPs do not necessarily lead to immune mediated injury and support the development of CMPs for clinical testing (29, 64). Bringing such vaccines to the treatment armamentarium may significantly improve outcomes for patients.

## Clinical Aspects of Mimicry

The potential benefits of inducing TACA-reactive antibodies in patients with cancer are demonstrated by observations that patient survival significantly correlates with ganglioside-reactive IgM levels (97). The fact that survival rates of cancer patients are correlated with low-titer, and presumably low-affinity, TACA-reactive antibodies argues that more robust antibody responses may not be necessary. Cross-reactions are important issues in vaccine the development field. As self-antigens induce tolerance, vaccination with non-self-antigens that molecularly mimic self-antigens may avoid tolerance and lead to generation of anti-tumor immune responses. In this context, little attention has been paid to the fact that the tumor-associated antigen MUC1 might be a natural CMP. In a series of studies from McKenzie’s group, it was noted that anti-α-Gal antibodies reacted with MUC1 antigens and that anti-MUC1 antibodies reacted with the α-Gal sugar (56). In mice, MUC1 peptide immunization resulted in cellular responses with reported little humoral response. In contrast, the MUC1 peptide induced a strong antibody response in human immunization. It was argued that pre-existing anti-Gal antibodies in human was the basis for the differential response as the Gal-epitope is a natural antigen in mice (56).

The mimicking of MUC1 with the Gal-epitope might have important consequences. The natural cross-reactivity of anti-Gal antibodies against MUC1 might lend to confusion making it difficult to ascertain the relative contributions of antibodies in binding to MUC1 upon MUC1 immunization; e.g., whether one can dissect if anti-MUC1 antibodies are not anti-TF and whether anti-MUC1 antibodies are not anti-Gal antibodies (98). On the other hand, the cross-reactivity of anti-Gal antibodies with MUC1 might lend to anti-MUC cellular immunity. Dendritic cells (DCs) play an important role in the induction of T-cell responses. Fc gammaRs (FcγR), expressed on DCs, facilitate the uptake of complexed antigen, resulting in efficient MHC class-I and MHC class-II Ag presentation and DC maturation (99, 100). IgG-complexed MUC1 internalized through FcγR on DCs might be are efficiently presented to CTLs through the MHC class-I pathway as observed in other systems (99, 100). However, these mechanisms might also responsible for antibody-mediated enhancement *in vivo* as suggested by the McKenzie work in humans and in animal models where antigen-IgG and IgE complexes exacerbated Th2 cells rather than Th1 cells (101). Therefore, mimicry of the Gal-epitope by MUC1 might skew Th2 type responses to MUC1 vaccines, which is contradictory to the present paradigm that stresses Th1 responses as being beneficial to MUC1 and other tumor-associated antigens.

While CMPs of TACA have been described that include the ganglioside GD2 (89, 95, 102–105), the ganglioside GD3 (106), sialylated Lewis a/x (107), and LeY (89), none of these CMPs have made it to the clinic except for our P10s. In contrast, several anti-idiotypic antibodies that mimic different GD2 (108–111), GD3 (112), and N-glycolyl (NGc) gangliosides (40) have made it to clinical trials. The most advanced is the GD3 mimicking antibody Bec2, which has been tested in a Phase III trial (112). Unfortunately, there was no improvement in survival or progression-free survival in the vaccination arm with Bec2. Each of these anti-idiotypes seems to have a different mechanism of action against cancer cells but parallel mechanisms observed with CMPs. In the case of the anti-idiotype that mimics NGc gangliosides it generates a humoral response that triggers cell death but differently than typical apoptosis (113). Patients that developed IgG and/or IgM Abs against NeuGcGM3 showed longer median survival times (114). Immunizations with the GD2/GD3 surrogates are less mechanism based. Bec2 induces antibody responses in about 25% of subjects (115). Consequently, different strategies using Bec2 have been considered including priming (38) and in combination therapy with adjuvant (116). For GD2, the anti-idiotypes induce GD2 reactive antibodies, which mediate ADCC activity. This type of data suggests that the anti-idiotypes generally generate IgG1 type antibodies are efficient at ADCC (108) while IgG2, which are considered carbohydrate reactive is minimal at mediating ADCC.

## Future Development of Structure-Based Vaccines

Glycans or TACAs are important targets for cancer immunotherapy as suggested by immune surveillance mechanisms. TACAs display important biological effects in tumor biology and tumor immunology. Most importantly, the recognition properties of glycans by immune effector cells have suggested translational strategies in immune therapy. In this review, we elaborated on achievements that facilitate rational vaccine design using CMPs. In nature, immunogenic parts of pathogens and cancer cells that provide antigens for B-cell receptors and antigenic peptides that are presentable by MHC molecules to TCR have to be identified. There is much to learn from the B/TCR that see carbohydrates as antigens and then as immunogens. Carbohydrates define recognition patterns, which activate the innate immune system to induce an appropriate adaptive immune response. Regular considerations in using CMPs that are selected upon binding to these receptors have not been pursued in the clinic with much fervor. This is partly due to the perception of utility and the idea that we need “specific” responses to singular carbohydrate antigens. More thought needs to be directed toward rational design approaches, which we have shown can be successfully implemented and not indiscriminate studies of co-crystallization or NMR studies with CMPs derived from random phage screening that are selected biased toward high-affinity binders (13–15).

While there are no universally accepted strategies and tools to rationally design vaccines to elicit antibody responses, vaccines should include B-cell receptor epitopes, but these might be more of a clustered type as we have shown using MAP platforms. Nanoparticle concepts could play a role here if they can be manufactured under GMP clinical grade. MHC Class-I/II molecules process Glycopeptides and so it is thought that these could be incorporated as well. The choice of carbohydrate might also impact on inducing Th1 or Th2 responses. We have shown that naked peptides can do the same however (63, 77). These glycopeptides or naked peptides should display sequences that allow T-cell epitope formation in a complex with MHC molecules but with the realization that there are hundreds of alleles that are differentially combined between individuals. Choosing immunogenic peptides presented by MHC faces the challenge of not only predicting sequences appropriate for complex with a particular MHC allele, but also finding peptides that can reliably build epitopes in the diverse genetic background within a human population.

The diversity of regulatory mechanisms involving glycans expands the range of possible effects of TACA targeting immunotherapeutic approaches (117). Anti-TACA antibodies, thus, may be involved in more than direct tumor cytotoxicity even though this mechanism is exciting. Although, the exact mechanism may represent a cascade of steps that are still to be established, TACA targeting has the potential to yield anti-tumor effects mediated by Natural Killer cells, which has not been thoroughly investigated in humans even though there is some evidence of therapeutic benefit (118, 119) or through neutralization of tumor immunosuppressive factors in the form of soluble gangliosides (120–122). Future work should clarify the points of involvement of antibody/carbohydrate interactions in modulating tumor growth and facilitating innate surveillance mechanisms.

The number of manuscripts published on CMPs has certainly diminished in recent years. The promise of CMPs ‘to be functional in animal models of bacterial infection has been impressive starting with our own (18), yet no CMP for bacterial antigens has made it to the clinic. The same is said for anti-idiotypes of bacterial carbohydrate antigens. In the cancer area only our P10s CMP has made it to the clinic. Perhaps the problem with diminished work on CMPs is more about the perception of mimicry rather than outcomes. The same has been suggested for anti-idiotypes (34). In mouse models, CMPs are functionally relevant much like anti-idiotypes. But clinically, all vaccine types, mimic or not, display less than optimal activity. The focus in immunotherapy has therefore been centered on checkpoints that mediate the immune response. Nevertheless, the promise of CMPs and other surrogates of carbohydrates is to better understand the structural implications of the antibody-mediated interactions that has the potential for innovation in terms of rational design of reagents with biological, chemical, and pharmaceutical applications that underlies concepts of reverse immunology which is highlighted herein.

## Conflict of Interest Statement

The authors declare that the research was conducted in the absence of any commercial or financial relationships that could be construed as a potential conflict of interest.
